# Anti-influenza A virus activity of two *Newtonia* species and the isolated compound myricetin-3-*o*-rhamnoside

**DOI:** 10.1186/s12906-021-03250-0

**Published:** 2021-03-16

**Authors:** Katlego E. Motlhatlego, Parvaneh Mehrbod, Fatemeh Fotouhi, Muna Ali Abdalla, Jacobus N. Eloff, Lyndy J. McGaw

**Affiliations:** 1 Department of Paraclinical Sciences, Phytomedicine Programme, Faculty of Veterinary Science, University of Pretoria, Pretoria, South Africa; 2 Present address: Department of Pharmacy and Pharmacology, Faculty of Health Sciences, University of the Witwatersrand, 7 York Road, Parktown, Johannesburg, 2193 South Africa; 3 Influenza and Respiratory Viruses Department, Pasteur Institute of Iran, Tehran, Iran

**Keywords:** Antiviral, Cytotoxicity, Diarrhoea, Fabaceae, Influenza a virus, *Newtonia*, Myricitrin

## Abstract

**Background:**

Some viruses play a key role in the disturbance of the digestive system. The common viruses which cause infectious diarrhoea (gastroenteritis) include astrovirus, caliciviruses, coronavirus and torovirus which are single-stranded RNA viruses. Influenza A virus (H1N1) also causes diarrhoea in addition to being associated with respiratory symptoms. In preliminary studies, *Newtonia hildebrandtii* and *N. buchananii* leaf extracts had good antibacterial activity against some bacteria implicated in causing diarrhoea. The aim of this study was to evaluate the anti-influenza activity of two *Newtonia* species extracts and the isolated compound (myricitrin).

**Methods:**

*N. hildebrandtii* and *N. buchananii* acetone, and MeOH: DCM (methanol-dichloromethane) leaf and stem extracts, and an antibacterial compound myricetin-3-*o*-rhamnoside (myricitrin), isolated from *N. buchananii,* were evaluated for their antiviral efficacy against influenza A virus (IAV) PR8/34/H1N1 as a model organism. The MTT and hemagglutination assays were used to assess the extracts and compound interference with cell viability and viral surface HA glycoprotein. The quantitative real-time PCR was performed to assess the viral load.

**Results:**

Plant extracts of *N. hildebrandtii* and *N. buchananii* were effective against IAV. The extracts in combination with H1N1 showed highly significant antiviral activity (*P* < 0.01) and maintained cell viabilities (*P* < 0.05). Myricitrin was non-cytotoxic at concentration 104 μg/ml. Myricitrin was most effective against IAV in a co-penetration combined treatment, thereby confirming the inhibitory effect of this compound in the viral attachment and entry stages. Myricitrin treatment also resulted in the highest viability of the cells in co-penetration treatment. The activity of myricitrin indicates the potential of the extracts in controlling viral infection at the attachment stage. The antiviral effect of myricitrin on IAV load in MDCK cell culture was confirmed using quantitative real-time PCR.

**Conclusion:**

Data from this study support further research and development on *Newtonia hildebrandtii, Newtonia buchananii* and myricitrin to address diarrhoea and related conditions caused by viruses in both human and veterinary medicine. Further work needs to be conducted on the activity of the extracts and the purified compound on other viruses of importance which have similar symptoms to influenza virus such as the coronavirus which led to a recent global pandemic.

## Background

In comparison with bacterial infections, viral infections are often difficult to treat, primarily because viruses spread and mutate rapidly [1]. Viral infections of the gastrointestinal tract (GIT) predominately manifest in immunocompromised patients [2]. Viruses are recognised as important causes of diarrhoea in adults and children and their pathogenesis is not completely understood [3–5]. This global problem is being actively investigated and continues to be extensively studied to improve epidemiology, understand the host immune response and develop vaccines [6, 7]. Influenza A virus, norovirus, sapovirus, adenovirus, rotavirus and astrovirus are the most common viral causes of diarrhoea [8–10]. Viral gastroenteritis outbreaks mostly occur in places such as nursing homes, health care institutions, schools and prisons [10]. Humans are susceptible to infections caused by viral diseases such as influenza A virus [11]. Influenza virus is characterised by its envelope with a negative-strand, eight-segmented RNA genome [1, 11]. Viruses can affect any part of the body. The main route of human influenza virus infection is through the respiratory and gastrointestinal tracts which cause diarrhoea, abdominal pain, vomiting and anorexia [12–14]. Influenza A virus replicates in human intestinal tissues and causes severe gastrointestinal symptoms [15–17]. The mechanism of the clinical significance and pathophysiology of influenza viruses in the gastrointestinal tract is unclear [13, 14, 17]. However, the interconnected relationship has been explained on the basis that the respiratory and intestinal systems have a common mucosal immune system [14]. The neuraminidase inhibitors such as oseltamivir and zanamivir, and M2 inhibitors such as amantadine and rimantadine are currently used to prevent and treat influenza infections [18–20]. Side effects such as neuropsychiatric events, headache, nausea, dizziness, vomiting, abdominal pain and diarrhoea brought about by the central nervous system and gastrointestinal tract during a viral infection, and the rapid emergence of antiviral resistance are realized where adamantanes are not quite effective in the prevention and treatment of influenza [12, 21]. Due to side effects of currently available medication, the use of plant-based antivirals against influenza virus may be promising [12, 21]. *Newtonia hildebrandtii* (commonly known as Lebombo Wattle) and *N. buchananii* (commonly known as Forest Newtonia) extracts have low cytotoxicity and good antimicrobial activity against diarrhoea-causing organisms [22]. Based on the high antimicrobial activity of *N. buchananii*, the plant was selected for isolation of antimicrobial compounds. Bioactivity guided isolation led to the isolation of myricitrin [23]. The compound was isolated by using chromatographic techniques and identified by means of 1D and 2D NMR spectroscopic and mass spectrometry analysis. Myricitrin was selected on the basis of purity and amount. As a result, only its structure was documented and considered for further research. The next step in the study of antidiarrheal efficacy of *Newtonia* species was to investigate whether the crude extracts from *N. hildebrandtii* and *N. buchananii* and the isolated antimicrobial flavonoid myricitrin from *N. buchananii* have antiviral activity against H1N1 which could be beneficial in combating diarrhoea.

## Methods

### Plant material and extraction

The plant species were collected from the Lowveld National Botanical Garden in Nelspruit, Mpumalanga, South Africa in summer months. One of the authors (Lyndy J. McGaw), a botanist identified the plant material and voucher specimens (PRU 122347 for *N. hildebrandtii* and PRU 122348 for *N. buchananii*) were deposited in the H.G.W.J. Schweickerdt Herbarium at the University of Pretoria (South Africa) for botanical authentication. The collected plant material was dried at room temperature in a well-ventilated room and ground to a fine powder in a Macsalab Mill (Model 2000 LAB Eriez). Ground dry powder (1 g) of each plant part (leaves and stems) was separately extracted in 10 ml of acetone and 1:1 MeOH-DCM (methanol-dichloromethane) (technical grade, Merck) in polyester centrifuge tubes. Different solvents were used including acetone, MeOH and DCM based on their polarity and to achieve the highest recovery yields. Additionally, this would also make sense for the bioactivity. The tubes were vigorously shaken for 30 min on an orbital shaker, then centrifuged at 4000 x g for 10 min and the supernatant was filtered through Whatman No.1 filter paper before being transferred into pre-weighed glass jars. This extraction procedure was repeated thrice on the same plant material and the solvent was removed by evaporation under a stream of air in a fume hood at room temperature to yield the dried extract. For isolation purposes, the same extraction method was followed with a slight modification where 300 g of *Newtonia buchananii* were extracted with 3000 ml of acetone (technical grade, Merck). The bottle was vigorously shaken and left overnight at room temperature and the supernatant was filtered through Whatman No.1 filter paper before it was transferred into pre-weighed glass containers. The extraction yield of *Newtonia hildebrandtii* extract was 23% with acetone and 28% with MeOH-DCM. While the yield of *Newtonia buchananii* was 5.67% with acetone and 11% with MeOH-DCM. The acetone crude extract was 43.8 g, the fraction with the bioactive compound was 18.1 g (which yielded an active subfraction 0.1522 g) and the isolated compound was 67.9 mg. Therefore, based on the formula the percentage yield of the isolated compound was 44.6% from the subfraction with the bioactive compound.

The extraction yield was calculated as follows:

$$ \mathrm{Plant}\ \mathrm{crude}\ \mathrm{extract}\mathrm{ion}\ \mathrm{yield}\ \left(\%\right)=\frac{\mathrm{Mass}\ \mathrm{of}\ \mathrm{dried}\ \mathrm{extract}\ \left(\mathrm{g}\right)\ }{\mathrm{Mass}\ \mathrm{of}\ \mathrm{plant}\ \mathrm{powder}\ \mathrm{extract}\mathrm{ed}\ \left(\mathrm{g}\right)}\times 100 $$

### Cytotoxicity assay

The cytotoxicity of the extracts against Madin-Derby Canine Kidney (MDCK) cells was determined by the MTT [3-(4,5-dimethylthiazol-2-yl)-2,5-diphenyltetrazolium bromide] reduction assay as previously described by Mosmann [24] with slight modifications [25]. MDCK cells were maintained at 37 °C and 5% CO_2_ in a humidified environment in Dulbecco’s Modified Eagle’s Medium (DMEM) (ICN, UK) and supplemented with 10% Fetal Bovine Serum (FBS) (Gibco, Gaithersburg, MD) and 1% Penicillin/ Streptomycin (Pen/Strep, Sigma Co.). The cells were seeded at a density of 3 × 10^5^ cells/ml (100 μl) in 96-well microtiter plates and incubated at 37 °C overnight to allow attachment and 80% confluency. Then, extracts dissolved in 100 μl of dimethyl sulfoxide (DMSO) at varying final concentrations were added to the cells. Doxorubicin hydrochloride (Pfizer) was used as a positive control. Wells made of cells in fresh medium without any treatment were used as negative control. Dimethyl sulfoxide as a vehicle control with maximum 0.5% concentration was tested as well.

The plates were further incubated at 37 °C and 5% CO_2_ for 48 h. After incubation, the medium was aspirated from the cells, which were then washed with phosphate-buffered saline (PBS). Then, 200 μl fresh medium together with 30 μl MTT (5 mg/ml in PBS) were added to each well and the plates were incubated at 37 °C in a 5% CO_2_ humidified incubator for 4 h. The medium was carefully aspirated from the wells and the resulting formazan crystals were solubilized by adding DMSO. The plates were placed on an orbital shaker for about 2 min. The absorbance was measured using a microplate reader (BioTek Synergy) at 570 nm. Cell growth inhibition for each extract was expressed in terms of CC_50_ values, defined as the cytotoxic concentration that caused 50% toxicity to cells. Then non-cytotoxic concentration (NCTC) values were calculated using SPSS. The tests were carried out in quadruplicate and each experiment was repeated thrice.

### Cell culture and influenza virus propagation

MDCK cells were grown in DMEM, supplemented with 10% FBS and 1% Penicillin/ Streptomycin (Pen/Strep, Sigma Co.) in a humidified incubator at 37 °C. The media was changed two to three times per week. The influenza vaccine strain, A/PR8/34 (H1N1), purchased from American Type Culture Collection (ATCC) with the Reference Number VR-897™, was propagated in MDCK cells in the presence of 1 μg/ml of Tosylamide Phenylethyl Chloromethyl Ketone-treated Trypsin (Trypsin_TPCK) (Sigma, USA) to create the working stock. For virus-stock preparation, the MDCK cells were infected with the virus at a multiplicity of infection (MOI) of 0.5. A standard HA test using cell culture infectious dose 50 (CCID_50_) and the Karber formula were conducted to measure virus infectivity [26].

### Anti-influenza effect of plant extracts and isolated compound

The amount of virus used in each experiment was based on the infected target cells of 0.5 MOI [27]. During antiviral evaluations, medium supplemented with FBS was removed and the cells were washed with PBS. Then media supplemented with Trypsin-TPCK was added. Influenza A virus (100 CCID_50_/0.1 ml) was added to the cells in combination with the extracts NCTC and incubated for 1 h at 37 °C. Following the incubation, unabsorbed viruses were washed and TPCK-containing medium (1 μg/ml) was added. Following 48 h incubation, the viability of the infected and non-infected cells was evaluated by MTT method. Meanwhile, the virus antigen titer in different treatments was determined by the hemagglutination assay (HA). To the 50 μl of the serially diluted virus solutions in U-bottomed 96-well plates (Nunc, Denmark), 50 μl of the chicken red blood cell (cRBC) suspension (0.5% volume in PBS) were added. After 60 min incubation, the endpoint of hemagglutination was evaluated by direct observation of RBCs precipitation [25]. Co-penetration procedure for 30 min was conducted for the extracts. Three types of treatments (co-, pre- and post-penetration) were used for the compound. In pre- and post-penetration procedures the virus was added to the cells after and before compound, respectively.

Amantadine hydrochloride (98.5 μg/ml) (Sigma, Saint Louis, Missouri, USA) was tested in parallel as control antiviral group. The cells without extract exposure (only media) served as negative control. Dimethyl sulfoxide as a vehicle control with maximum 0.5% concentration was tested as well. The abiotic control of the extracts and compound diluted in medium with no cells was included to nullify any interference in the assay due to absorption of light by extract itself.

### Molecular evaluation of myricitrin antiviral effect

#### RNA extraction and cDNA synthesis

The cell supernatants of all treatments were exposed to viral RNA extraction by High Pure Viral Nucleic Acid Kit (Roche, Germany) according to the manufacturer’s instruction. Briefly, the cell culture supernatant was mixed with Binding Buffer (BB) supplemented with poly A and proteinase K and incubated at 72 °C for 10 min. The lysate mixture was transferred to a column placed in a 2 ml collection tube and centrifuged at 8000 x g for 1 min. The Inhibitor Removal Buffer (IRB) was added to the column and centrifuged. Two-step washing and centrifugation (13,000 x g for 1 min) was then conducted to dry the column matrix. Then Elution Buffer (EB) was added and centrifuged (8000 x g for 1 min). Aliquots of the eluted RNA were stored at − 80 °C.

All RNA samples (10 μl) were subjected to cDNA synthesis using Transcriptor First Strand cDNA Synthesis kit (Roche, Germany) including 5X Transcriptor Reverse Transcriptase Reaction buffer, random hexamer primers, Protector RNase Inhibitor, dNTP mix and Transcriptor Reverse Transcriptase in a final volume of 20 μl. The mix was incubated at 25 °C for 10 min followed by 55 °C for 30 min and terminated at 85 °C for 5 min. The synthesized cDNAs were stored at − 20 °C. The concentration of the cDNA templates was measured using the Picodrop Spectrophotometer system (Alpha, Biotech, UK). Virus-inoculated and mock-infected samples were considered as positive and negative controls, respectively.

#### Quantitative real-time PCR

The Real-time PCR reactions were performed using LightCycler FastStart DNA Master SYBR Green I (Roche, Germany) and InfA M2-A-For (GAC CRA TCC TGT CAC CTC TGA C 3′) and InfA M2-A-Rev (5′ AGG GCA TTY TGG ACA AAK CGT CTA 3′) primers to produce an amplicon of 130 bp covering nucleotides 146 to 276 using Corbett Rotor-Gene Q 6000 (Corbett Research, Australia) in a total volume of 20 μl. All materials were mixed and prepared in 0.2 ml PCR tubes in the dark. The thermal cycling program was performed using a three-step cycling protocol **(**Table 1**)**. The copy number in each treatment was calculated by the following formula [28]:

$$ \mathrm{Number}\ \mathrm{of}\ \mathrm{copies}/\mu \mathrm{l}=\left[6.02\times 1{0}^{23}\ \left(\mathrm{molecules}/\mathrm{mole}\right)\times \mathrm{DNA}\ \mathrm{concentrations}\ \left(\mathrm{g}/\mu \mathrm{l}\right)\right]/\left[\mathrm{Number}\ \mathrm{of}\ \mathrm{base}\ \mathrm{pairs}\times 660\mathrm{D}\right] $$

**Table 1:** PCR protocol for M2 gene amplification

Step	Temperature (°C)	Time	Number of cycles
Initial denaturation	95	10 min	1
Denaturation	95	10 s	35
Annealing	55	10 s	
Extension	72	30 s	
Final elongation	72	5 min	1

That 6.02 × 10^23^ (molecules/mole) is Avogadro’s number and 660 D is average weight of a single base pair.

### Statistical analysis

The data expressed as mean ± SD was analyzed by analysis of variance (ANOVA) (SPSS 18.0), Tukey post-hoc test. Sample values with *P* < 0.05 were considered significant.

## Results

### Cytotoxicity assay results

The CC_50_ and NCTC of the extracts were obtained using the MTT cytotoxicity assay and SPSS analysis as shown in Table 2. The NCTC of myricitrin was obtained in a similar method as mentioned for the extracts and this compound was not cytotoxic at 104 μg/ml.

**Table 2:** Cytotoxicity of the plant extracts normalized to negative control

Plant name	Plant part	Extracts	CC_**50**_ (mg/ml) Mean ± SD	NCTC (mg/ml) Mean ± SD
*Newtonia hildebrandtii*	Leaves	Acetone	0.170 ± 0.036	0.090 ± 0.008
	Leaves	MeOH: DCM	0.031 ± 0.005	0.017 ± 0.002
	Stem	Acetone	0.074 ± 0.003	0.034 ± 0.004
	Stem	MeOH: DCM	0.150 ± 0.050	0.077 ± 0.003
*Newtonia buchananii*	Leaves	Acetone	0.171 ± 0.031	0.088 ± 0.003
	Leaves	MeOH: DCM	0.749 ± 0.001	0.377 ± 0.004
	Stem	Acetone	0.119 ± 0.012	0.059 ± 0.001
	Stem	MeOH: DCM	0.087 ± 0.002	0.046 ± 0.004

*CC*_*50*_ cytotoxic concentration 50%, *NCTC* non-cytotoxic concentration, *MeOH* methanol, *DCM* dichloromethane

Amantadine hydrochloride CC_50_ and NCTC values were obtained at 197 ± 1.5 and 98.5 ± 0.0, respectively.

### Anti-influenza activity of extracts and compound

**Fig. 1:**
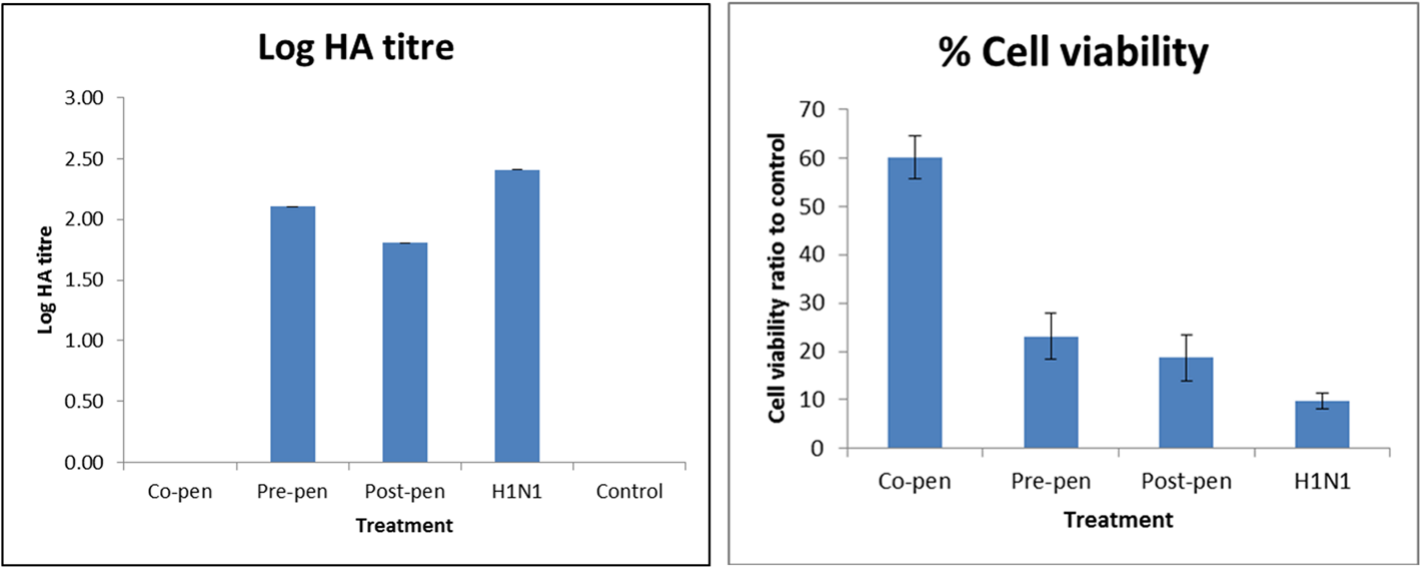
Log HA titer (left) and cell viability (right) of the combined treatments of myricitrin and H1N1 compared to H1N1 alone. The stars show the significant level between the values of combined treatments and H1N1; *: significant, **: highly significant

Based on the HA results (shown in Table 3**)**, plant extracts of *N. hildebrandtii* and *N. buchananii* were significantly effective against influenza H1N1 (*P* < 0.01). Increased cell viabilities in the combined treatments of extracts and H1N1 compared to the positive control (H1N1 alone) were markedly significant (*P* < 0.05). Myricitrin was not toxic to the cells at the highest concentration tested (104 μg/ml). Based on the cell viability results, myricetin-3-*O*-rhamnoside treatment led to the highest viability of the cells in the co-penetration treatment (Fig. 1).

**Table 3:** Log HA titer and cell viability results of the combined treatments of *Newtonia* species extracts and isolated compound compared to H1N1 only

Treatment		Log HA titre	Cell Viability (ratio to control)
H1N1 with no extract	Virus inoculation	2.408	0.169 ± 0.010
Extracts combined with H1N1	Nh1	0**	0.887 ± 0.111*
	Nh2	0**	0.860 ± 0.165*
	Nh3	0**	0.683 ± 0.078*
	Nh4	0**	0.679 ± 0.032*
	Nb1	0**	0.684 ± 0.051*
	Nb2	0**	0.719 ± 0.026*
	Nb3	0**	0.729 ± 0.012*
	Nb4	0**	0.711 ± 0.014*
Compound in co-penetration treatment with virus	J2S	0**	0.601 ± 0.043*

*N. hildebrandtii* acetone leaf extract (Nh1), *N. hildebrandtii* Methanol-DCM leaf extract (Nh2), *N. hildebrandtii* acetone stem extract (Nh3) *N. hildebrandtii* Methanol-DCM stem extract (Nh4), *N. buchananii* acetone leaf extract (Nb1), *N. buchananii* Methanol-DCM leaf extract (Nb2), *N. buchananii* acetone stem extract (Nb3) *N. buchananii* Methanol-DCM stem extract (Nb4), J2S (isolated myricetin-3-*O*-rhamnoside)

The stars show the significant level between the values of combined treatments and H1N1; *: significant, **: highly significant

### Molecular evaluation results

**Fig. 2:**
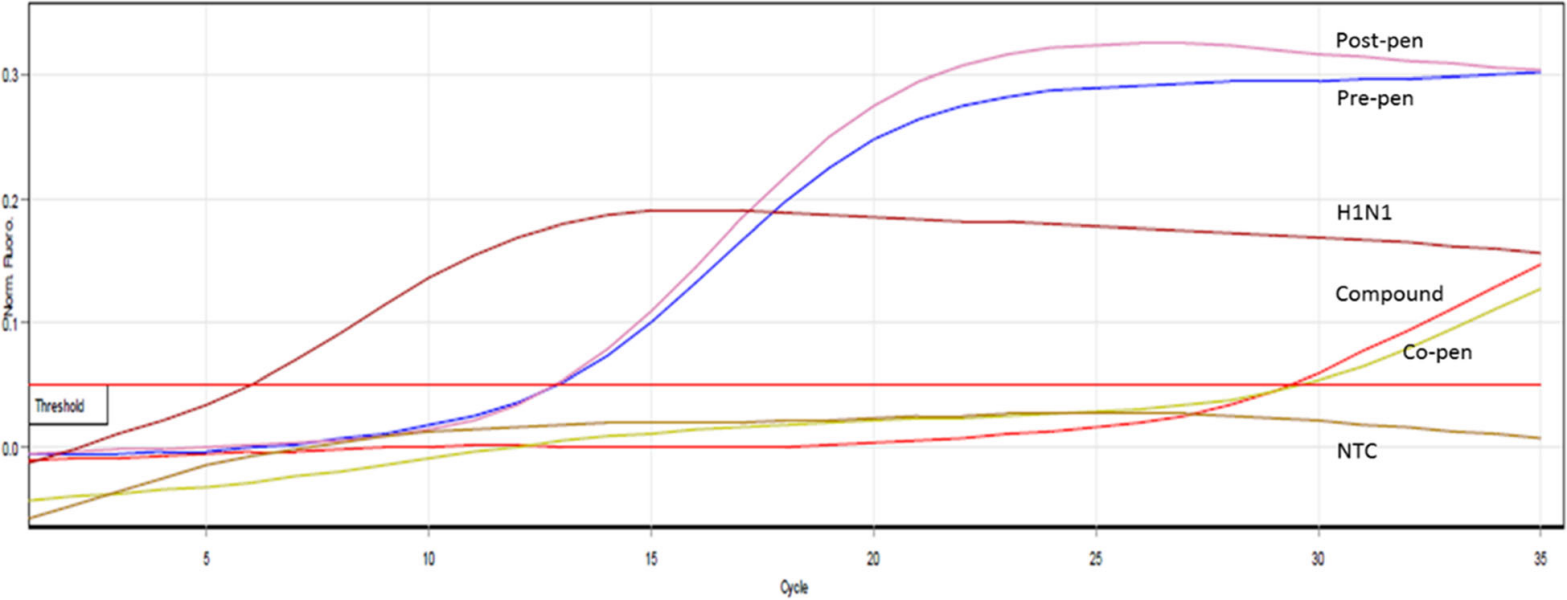
Amplification curves for M2 gene of H1N1 in all combined treatments of myricitrin with H1N1 compared to H1N1 alone

The amplification curves were generated by plotting cycle threshold values (Ct) on the X-axis against input cDNA concentration (copies/μl) on the Y-axis alongside a non-template control (NTC) to show the accuracy of the PCR process (Fig. 2).

The M2 log_10_ copy numbers after different combined treatments of compound and H1N1 were calculated based on the obtained Ct values and the related formula. Data shown in Table 4 verified the significant decrease (*P* < 0.01 & *P* < 0.001) in H1N1 log_10_ copy numbers in all combined treatments, especially the co-penetration treatment.

**Table 4:** M2 gene cycle thresholds, concentrations and log_10_ copy numbers following combination treatments with myricitrin

Treatment	Ct value	Concentrations (g/μl)	Log_**10**_ copy number
H1N1	5.97	**7.31 × 10**^**−7**^	**51 × 10**^**11**^
Co-penetration	29.37	**1.20 × 10**^**− 7**^	**8.4 × 10**^11***^
Pre-penetration	12.87	**6.12 × 10**^**− 7**^	**43 × 10**^11**^
Post-penetration	12.83	**6.14 × 10**^**−7**^	**43 × 10**^11**^

Note: M2 Log_10_ copy numbers relating Ct values during amplification of influenza A H1N1. **, ***: significant decrements (*P* < 0.01 & *P* < 0.001) in combined treatments in comparison with the virus untreated sample (H1N1)

## Discussion

There are different types of viruses which may cause gastrointestinal disorders especially diarrhoea such as norovirus, adenovirus, rotavirus, sapovirus, astrovirus and influenza A virus [8–10]. Viral infections are more difficult to treat than bacterial infections. Compounds from natural sources have become a subject of interest in controlling viral infections in modern society as natural alternatives to chemical medicines [29]. They may act directly against viral infection or act through stimulating the body immune system [29, 30]. Flavonoids often have antiviral activity [31], with protective effects against bacterial and viral infectious diseases [32]. Although medicinal plants have been used for different remedies, the safety of the crude extract is questionable as some phytochemicals may exist at toxic levels [33]. Therefore, focusing on active pure compounds is of great importance.

On the basis that diarrhoea is caused by both bacterial and viral infections, the effect of the *Newtonia* extracts and isolated flavonoid compound was investigated against influenza virus A/PR8/34 (H1N1) as a model organism. *Newtonia hildebrandtii* and *N. buchananii* acetone and MeOH: DCM leaf and stem extracts, and myricitrin compound were evaluated for antiviral mechanism of action against H1N1 strain of influenza as a model. For the extracts, only co-penetration viral treatment was performed and this was done just to see if the extract has the ability to inactivate the virus. The isolated antimicrobial compound, myricitrin, was tested in pre-, co- and post-penetration experiments.

The significant increase in cell viabilities as compared to the cells infected with H1N1 alone demonstrated the protective effect of extracts on cell viability against viral cytopathic effects. The extracts in combination with the H1N1 showed highly significant antiviral activity on viral titer (*P* < 0.01) and maintained cell viabilities (*P* < 0.05).

The HA was also used to evaluate how myricitrin interferes with viral attachment. This compound was not toxic to cells at the highest concentration tested (104 μg/ml) and was effective against influenza virus in the co-penetration combined treatment **(**Table 3**)**, supporting the inhibitory effects of the antibacterial compound in the viral attachment and entry stage.

The outcome motivated consideration of the antiviral activity of myricitrin at molecular level. The antiviral effect of myricitrin on influenza A virus load in MDCK cell culture was analyzed using quantitative real-time PCR assay **(**Table 4**)**. The absolute quantification calculation determined the substantial copy numbers from the target gene related to the Ct values. The log_10_ copy numbers which were calculated from the concentrations against mean Ct values, confirmed significant differences between H1N1 PCR product and combined-treated PCR products. The significant increments in cycle thresholds (Cts) of M2 PCR products were shown (*P* < 0.01 & *P* < 0.001) once the compound was applied in all types of combined treatments especially in the co-penetration treatment. The data obtained from molecular assay confirmed the HA result which showed HA decrement in combination treatments.

Binding of the extracts and isolated compound (myricitrin) to the viral envelope glycoprotein of H1N1 blocked the interaction with cell receptors and inhibited infection. The significant increase in cell viabilities in combined treatments compared to H1N1 infection alone demonstrated the protective effect of extracts and myricitrin on the cell viability against viral cytopathic effects. The *Newtonia* species have potential to reduce the complications caused by viral infections responsible for diarrhoeal symptoms. The acetone and MeOH: DCM leaf and stem extracts of *N. hildebrandtii* and *N. buchananii* species which both were effective against viral load with no cytotoxic effect on cell viability have the capacity for further investigation as a source of new therapeutic compound development against H1N1 and other viruses.

## Conclusion

To the best of our knowledge no antiviral research has been conducted on the studied plants. Myricitrin, a flavonoid compound purified from *N. buchananii,* had anti-influenza H1N1 activity in this study, and this was confirmed by investigating the genome load of the virus using quantitative real-time PCR assay. Myricitrin can be considered as an effective compound against influenza viral infection as both prophylaxis and treatment, and has the potential as an HA- inhibitor drug. In vivo studies are suggested to be conducted in the future to confirm this potential. *N. hildebrandtii* and *N. buchananii* extracts have the potential to treat and prevent diarrhoeal infections because of the antibacterial and antibiofilm activities [34]. This study added more value to these species in demonstrating their potential efficacy in treating diarrhoea caused by viral episodes. These plants, as well as myricitrin, are therefore recommended for further testing and development into herbal medicines or remedies useful in the management of viral infections. It is worthy for further evaluation of the antiviral efficacy of these extracts and especially the active compound against other viral diseases with similar or even more severe symptoms that may cause epidemics and pandemics such as SARS-CoV-2 which has shown several symptoms like flu including respiratory and gastrointestinal symptoms.

## Data Availability

The datasets used and/or analyzed during the current study are available from the corresponding author on reasonable request.

## Abbreviations

MIC: Minimum inhibitory concentration
MeOH: Methanol
DCM: Dichloromethane
MTT: 3-(4,5-dimethylthiazol-2-yl)-2,5-diphenyltetrazolium bromide
INT: P-iodonitrotetrazolium violet
DMSO: Dimethyl sulfoxide
PBS: Phosphate-buffered saline;
DMEM: Dulbecco’s Modified Eagle’s Medium
LC_50_: 50% lethal concentration,
HA: Hemagglutinin
NA: Neuraminidase
M2: Matrix protein 2
